# Sufficient and necessary conditions for ChatGPT adoption in medical education: a combined partial least square-structural equation modelling and necessary condition analysis

**DOI:** 10.1186/s12909-026-09244-1

**Published:** 2026-04-29

**Authors:** Keng Sheng Chew, Shirly Siew-Ling Wong, Say Keat Ooi, Zhi Jun Lim, Gregg Griffin Jensen, Nur Nayli Zulaikha Khairul Azri, Latchumy Shanmuganathan

**Affiliations:** 1 Faculty of Medicine and Health Sciences, Universiti Malaysia Sarawak, Kota Samarahan, Sarawak 94300 Malaysia; 2 Faculty of Economics and Business, Universiti Malaysia Sarawak, Kota Samarahan, Sarawak 94300 Malaysia; 3 Graduate School of Business, Universiti Sains Malaysia, USM Pulau Pinang, Gelugor, Penang 11700 Malaysia

**Keywords:** ChatGPT, Behavioral intention, Technology acceptance, Structural Equation Modelling, Necessary Condition Analysis

## Abstract

**Background:**

ChatGPT has gained rapid adoption in education due to its capacity to generate human-like responses, support personalized learning, and assist with complex knowledge retrieval. Yet, concerns about misinformation, overreliance, privacy, and ethical risks continue to shape students’ acceptance of AI tools. While prior research on factors influencing ChatGPT adoption has relied on regression-based approaches using frameworks like the Technology Acceptance Model, these methods assess only the sufficiency but not the necessity of these factors. This study combined Partial Least Squares Structural Equation Modelling (PLS-SEM) with Necessary Condition Analysis (NCA) to determine both the sufficient and necessary factors influencing medical students’ intentions to use ChatGPT for learning.

**Methods:**

A cross-sectional survey was conducted among 146 pre-clinical Year 2 medical students at Universiti Malaysia Sarawak (UNIMAS). Seven variables were measured, i.e., perceived usefulness (PU), perceived ease of use (PEOU), social influence (SI), hedonic motivation (HM), perceived risk (PR), attitude (At), and behavioral intention (BI). Data were analyzed using SmartPLS 4.0, following a two-stage SEM approach, and NCA using the ceiling envelopment–full disposal hull (CE-FDH) technique.

**Results:**

PLS-SEM showed substantial explanatory power (R² = 0.68) with attitude (β = 0.600), social influence (β = 0.133), and perceived usefulness (β = 0.096) as significant factors influencing intention for ChatGPT adoption. NCA revealed that attitude (d = 0.384), social influence (d = 0.310), and perceived usefulness (d = 0.183) were both necessary and sufficient conditions for BI whereas, hedonic motivation (d = 0.235) and perceived risk (d = 0.234), although non-significant in SEM, were significant necessary conditions, indicating threshold requirements of these 2 factors for adoption.

**Conclusion:**

By combining PLS-SEM and NCA, this study enhanced the richness and granularity of understanding on the factors shaping ChatGPT adoption in medical education.

**Supplementary Information:**

The online version contains supplementary material available at 10.1186/s12909-026-09244-1.

## Introduction

ChatGPT is a generative artificial intelligence (AI) tool that has gained rapid adoption in education due to its capacity to generate human-like responses that can be effective in supporting personalized learning [1]. In medical education, it has been increasingly used for generating clinical case scenarios, providing study support as well as for rapid retrieval of complex medical information [1, 2]. Nonetheless, despite the growing recognition of the benefits of ChatGPT in education including practical skills acquisition [1, 3], pockets of resistance persist due to lingering concerns about overreliance, diminished critical thinking, ethical risks such as misinformation, privacy breaches, and academic dishonesty [1, 3, 4]. Furthermore, unlike in many other fields, AI integration in medical education is uniquely complex as unchecked inaccurate or biased outputs can potentially misguide clinical reasoning resulting in downstream negative implications for patient safety, professionalism, and misinformation-related ethical risks [1, 2].

Recent studies using the Technology Acceptance Model (TAM) and the Unified Theory of Acceptance and Use of Technology (UTAUT) models have examined students’ intentions to adopt generative AI tools by focusing on factors such as perceived usefulness, ease of use, and social influences [5–7]. A review of these studies shows that many researchers have extended their TAM and UTAUT frameworks beyond their core constructs to include additional contextual factors such as trust [8–10], privacy and security concerns [8], perceived risk and anxiety [11–14], feedback quality [15] and design or interactivity features [11] (see Table 1). These findings suggest that generative AI adoption can be shaped by a broader range of extrinsic factors beyond the conventional TAM/UTAUT constructs to reflect the complex socio-psychological environment where ChatGPT is used in education.

**Table 1:** Summary of key studies on ChatGPT adoption in teaching and learning using TAM, UTAUT, and UTAUT2 frameworks

Study (year)	Country	Population & sample	Model & added variables	Statistical analysis	Key findings
Albayati [8] (2024)	Multi-country (online survey)	Undergraduate students, *N* = 603	Extended TAM with 4 additional variables: Privacy, Security, Social Influence, Trust	PLS-SEM	PEOU, PU, trust and social influence significantly influence attitudes. Security concerns significantly influence both PEOU and PU. Privacy significantly influence PU but not PEOU.
Liu et al. [11] (2024)	China	Medical students, *N* = 311	Extended TAM with 2 additional variabless: Perceived Risk and Social Impact	CB-SEM	Attitude significantly influenced intention and mediated the effects of PU, PEOU and PR. PR and SI significantly influenced BI.
Bhat et al. [9] (2024)	India	Higher education educators, *N* = 1,214	Extended UTAUT with the additional variable of Trust	SEM	Performance expectancy, effort expectancy, facilitating conditions, HM, and habit significantly influenced BI. Trust moderated the relationship between BI and actual adoption.
Budhathoki et al. [13] (2024)	Nepal and United Kingdom	University students, Nepal *N* = 239; UK *N* = 226	Extended UTAUT with the additional variable of Anxiety	SEM	Performance expectancy, effort expectancy, and social influence significantly influenced BI in both Nepal and the UK. Anxiety had no significant effect in Nepal but significantly reduced intention in the UK, reflecting cultural differences and stronger academic integrity concerns in UK.
Sallam et al.[12] (2023)	Jordan	Health and medical students, *N* = 458	TAM-based instrument (TAME-ChatGPT)	EFA and reliability testing	PR, PU, and PEOU, and attitude significantly influenced adoption in medical education.
Almogren et al. [15] (2024)	Saudi Arabia (with international collaboration)	Higher education students, *N* = 458	Extended TAM model with additional variables of Feedback Quality, Assessment Quality, Subjective Norms	SEM	PEOU and attitude significantly influenced BI. PU, feedback quality, and perceived assessment quality significantly influenced attitude.
Salifu et al. [10] (2024)	Ghana	Economics students, *N* = 306	Extended UTAUT2 model with the additional variables of Design, perceived Interactivity and perceived Trust	Hybrid SEM-ANN	Design and interactivity significantly influenced perceived trust, which in turn significantly influenced BI. SI, performance expectancy, HM, and habit significantly influenced BI. BI and facilitating conditions significantly influenced actual usage.
Alqaisi et al. [14] (2025)	Jordan	Medical students and faculty, *N* = 127	Modified UTAUT with additional variable of Perceived Risk	SEM	Performance expectancy (PE) and effort expectancy (EE) significantly influenced attitude (ATT), which in turn predicted behavioral intention (BI) and actual usage. Facilitating conditions did not significantly affect EE or BI, indicating low dependence on external support. Perceived risk (PR) did not negatively influence attitude.

Despite these contributions, most of these recent studies relied exclusively on sufficiency-based, net-effect modelling (e.g., using structural equation modelling or SEM) [16], to evaluate the factors that increase behavioral intention. However, this approach cannot determine whether any of these factors function as the “must-have” determinants, i.e., minimum conditions that must be met before high intention can occur, regardless of the strength of other predictors. By contrast, necessity logic works differently from sufficiency logic. Necessity logic focuses on conditions that are essential, or “must-haves”; without which, the outcome cannot occur, no matter how strong the other factors are.

According to Dul [17], necessary conditions function as “bottlenecks” or constraints that set the minimum requirements for an outcome to occur. For example, many medical schools require applicants to demonstrate a minimum level of English language proficiency to gain admission. While meeting this criterion alone does not guarantee admission, failure to meet it will result in rejection, regardless of the strength of other qualifications. Similarly, in medical education, overlooking these non-negotiable “must-have” bottlenecks can be harmful because adoption may proceed in ways that undermine safe clinical learning. For example, if students use ChatGPT without meeting a minimum threshold of risk mitigation (e.g., awareness of hallucinations and verification of information), inaccurate outputs may be accepted as correct and later carried into clinical reasoning.

Analyses of these necessary conditions (known as Necessary Condition Analysis or NCA) has been formally developed to identify and quantify such constraints [16, 17]. By combining sufficiency logic (using regression-based analyses) with necessity logic (using NCA), researchers can gain a fuller picture of causality, i.e., determining not only which factors that can significantly improve outcomes, but also which ones are indispensable [16, 17].

Based on TAM model, this study is believed to be among the first in medical education to combine Partial Least Square-Structural Equation Modelling (PLS-SEM) with NCA, with the aim to determine both the sufficient factors and the necessary factors that influence medical students’ behavioral intention to use ChatGPT for learning. While PLS-SEM can identify how strongly factors such as perceived usefulness, ease of use, and social influence shape behavioral intention, NCA can reveal whether certain factors or conditions act as the bottlenecks or “non-negotiable” prerequisites, without which the intention is not likely to occur, regardless of the strengths of the influence of other factors [17].

## Methods

This study employed a quantitative, cross-sectional survey design among pre-clinical Year 2 medical students at Universiti Malaysia Sarawak (UNIMAS), Malaysia. Ethics approval was obtained from our institutional UNIMAS Human Research Ethics Committee (HREC) (with approval no HREC(NM)/2023 (2)/71) prior to the commencement of this study.

This study was built primarily on Liu et al. (2024), which used a TAM-based model to determine medical students’ acceptance of large language models, making it the closest empirical basis for our medical education context. As mentioned, most prior ChatGPT adoption studies in education rely on regression-based analyses (e.g., SEM/PLS-SEM), that determined the significant causal relationships among variables but cannot determine whether any variable functions as non-negotiable necessity for significant behavioral intentions (BI). Mathematically, regression-based analysis follows the additive, sufficiency-based logic, $Y=a+b_1X_1+b_2X_2+b_3X_3+\dots$) where $a$ represents the intercept (constant term) and each $b_1$ denotes the regression coefficient indicating the expected change in the dependent variable $Y$ for every one-unit change in the corresponding independent variable $X_1$. On the other hand, necessity logic can be expressed as a bottleneck, multiplicative framework, $Y\;=\;X_1\;\times\;X_2\;\times\;X_3\;\times\;\dots$) where a zero level in any necessary condition constrains the outcome.

To address this methodological gap, we integrated PLS-SEM with Necessary Condition Analysis (NCA) to determine both statistically important variables influencing BI as well as the “must-have” necessary conditions. For the PLS-SEM analysis, the following hypotheses were specifically tested:

- H1: Attitude (At) positively influences BI to use ChatGPT for learning.
- H2: Hedonic motivation (HM) positively influences BI.
- H2: Hedonic motivation (HM) positively influences BI.
- H4: Perceived risk (PR) negatively influences BI.
- H5: Perceived usefulness (PU) positively influences BI.
- H6: Social influence (SI) positively influences BI.
- H7: Hedonic motivation (HM) positively influences attitude (At).
- H8: Perceived ease of use (PEOU) positively influences At.
- H9: Perceived risk (PR) negatively influences At.
- H10: Perceived usefulness (PU) positively influences At.
- H11: Social influence (SI) positively influences At.

### Participants

The entire cohort of 148 Year 2 medical students in the academic year 2024/2025 was invited to participate voluntarily, with anonymity ensured by omitting any personal identifiable information. This cohort of students was deliberately selected because they represent a cohort transitioning from foundational biomedical sciences to applied clinical learning within our two-phase undergraduate medical curriculum: Phase 1 (Years 1–2) focuses on basic biomedical sciences, while Phase 2 (Years 3–5) is clinical studies. Hence, the Year 2 students were believed to be more likely to use generative AI to clarify difficult concepts, summarize content, and link basic science to early clinical thinking. On the other hand, Year 1 students were excluded because we believe most of them are still adjusting to medical school learning demands particularly in the first semester, and including multiple year groups would introduce more heterogeneity that could confound the findings. Prior informed consent was obtained before the face-to-face administration of the questionnaires.

An a priori sample size estimation was conducted using G*Power (version 3.1, Mac) on a fixed-model multiple linear regression (test of $\text R^2$ deviation from zero), assuming α = 0.05, power (1-β) = 0.80, moderate effect size f² = 0.15 with 6 predictors or independent variables, to ensure that the sample size had adequate statistical power to detect the expected effect. Based on this calculation, the minimum required sample size was 146.

### Materials

The instrument was primarily adapted from the validated questionnaire used by Liu et al. [11], as it is the only study among the 8 reviewed studies that focus exclusively on medical students and is therefore most comparable to our context. Hedonic motivation (HM) was added because it has been shown to be an important factor of ChatGPT adoption in prior studies [9, 10], and its items were adapted from Bhat et al. [9]. In contrast, we did not include trust, privacy, and security as separate factors because these concerns represent closely related facets of perceived risk that is already captured in Liu et al. [11], and including these additional factors could introduce redundancy and multicollinearity. No additional pilot testing or formal content-validity exercise was conducted prior to adaptation; instead, reliability, convergent validity and discriminant validity was evaluated as part of the PLS-SEM procedures as described below.

Eventually, the data in our study were collected using a self-administered questionnaire on these 7 variables: PU, PEOU, SI, HM, PR, At, and BI to use ChatGPT for educational purposes. The detailed description of each item of the instrument used in this study is given as Supplementary File 1.

ChatGPT was selected because, at the time of data collection, it was the dominant and believed to be the most commonly used generative AI tool among students in our local setting. While other tools (e.g., Google Gemini, NotebookLM, Perplexity AI) could have been included, they were not as widely adopted locally at that time and comparing multiple platforms would add unnecessary variability due to differences in features and familiarity.

### Procedures

Data were collected using self-administered, printed questionnaires distributed in class and retrieved on the same day after completion. A census approach of the entire accessible cohort was applied by inviting all 148 Year 2 medical students in 2024/2025 to participate. A priori G*Power analysis was also performed to confirm that the sample size achieved was adequate for the planned model testing. Responses were then keyed into a spreadsheet.

Both PLS-SEM and NCA were performed using SmartPLS for Windows version 4.0 [18]. For PLS-SEM, the analysis was performed using the 2-stage approach by Anderson & Gerbing [19]. In the first stage, i.e., the measurement modelling stage, confirmatory factor analysis was performed. Specifically, convergent validity was assessed using factor loadings (with the cut-off > 0.7) and/or average variance extracted or AVE (with cut-off > 0.5) [20]. Discriminant validity was examined using the Fornell–Larcker criterion [21], heterotrait-monotrait ratio of correlations (HTMT) [22], and cross-loading. The HTMT criterion was utilized to determine discriminant validity in two ways. As a rule of thumb, HTMT values < 0.85 were interpreted as evidence of discriminant validity under a more conservative criterion or while values below 0.90 were considered acceptable under a more lenient criterion. Secondly, discriminant validity was also supported when the 95% bias-corrected, bootstrapped confidence intervals of the HTMT did not include the value of 1.00 [22].

Internal consistency reliability was evaluated using Cronbach’s alpha (with cut-off > 0.7) and composite reliability (with cut-off > 0.6) [23]. In the second stage, i.e., structural modelling stage, hypotheses evaluation was conducted. Bootstrapping with 500 re-samplings was performed. Inner model collinearity was assessed using variance inflation factors (VIF). VIF < 5 was interpreted to indicate no critical multicollinearity among the variables [20]. The model fit of our conceptual framework was determined using R² coefficients and t-statistics [20], whereby R² values of 0.02, 0.13, and 0.26 are interpreted as weak, moderate, and substantial levels respectively and the effect size f^2^, where 0.02, 0.50 and 0.35 denotes weak, moderate and substantial effect sizes respectively [20]. The predictive power of the model was assessed using the root mean squared error (RMSE) in the PLSpredict procedure [24].

For NCA, the ceiling line was determined using the ceiling envelopment (CE) with full disposal hull (CE-FDH) technique. Ceiling line is the line that separates the empty space without observations (i.e., the ceiling zone, C) from the space containing observations. The ceiling envelopment (CE) method, which is a non-decreasing stepwise linear approach indicated for discrete, ordinal, or dichotomous data, was used in this study instead of ceiling regression (CR) method (which uses ordinal linear regression function to smoothen the CE line), indicated for continuous data. This is because all items in this study were scored using an ordinal Likert-scale. The full disposal hull (FDH) approach was chosen over varying return to scale (VRS), as FDH does not assume line convexity and is considered more flexible [17].

The statistical significance of the effect size (d) of the latent variable scores on the dependent variable, BI, was determined using a permutation of 10,000 samples as recommended by Dul [17] where 0 < d < 0.1 = small; 0.1 ≤ d < 0.3 = medium; 0.3 ≤ d < 0.5 = large; and d ≥ 0.5 = very large. Conceptually, d represents the proportion of C relative to the total space where observations are possible (i.e., the scope, S); hence, d = C/S. A larger ceiling zone indicates a stronger constraint of the independent variable exerts on the dependent variable. A threshold of d ≥ 0.1 was used as the cut-off value to identify necessary conditions [16].

## Results

A total of 146 out of 148 Year 2 medical students (response rate 98.6%) participated in this study. Mean age was 21.11 (standard deviation +/- 0.44) years old. Out of these, 46 (31.5%) students were male students.

The measurement model demonstrated acceptable convergent validity and internal consistency reliability (Table 2), with all indicator loadings being statistically significant based on bootstrapped p-values. Although three indicators (PR2, PU2, and SI1) had loadings slightly below the preferred 0.70 threshold, they were retained because their respective constructs maintained adequate AVE (> 0.50) and the items were considered conceptually important for content validity in this study. Good discriminant validity was similarly demonstrated as no significant cross loading was observed, Fornell–Larcker criterion fulfilled as shown in Table 3 and the HTMT analysis in Table 4. Although two HTMT values exceeded the conservative 0.85 threshold and one marginally exceeded 0.90, all bootstrapped confidence intervals remained well below 1.00, indicating that the variables are empirically distinct [22].

**Table 2:** Convergent validity and internal consistency reliability of the data

Variable		Outer model		Average variance extracted (AVE)	Composite reliability (ρA)	Cronbach’s alpha
		Factor Loadings	p-value			
Attitude	At1	0.850	< 0.001	0.744	0.886	0.885
	At2	0.876	< 0.001			
	At3	0.882	< 0.001			
	At4	0.842	< 0.001			
Behavioral Intention	BI1	0.932	< 0.001	0.871	0.852	0.851
	BI2	0.934	< 0.001			
Hedonic motivation	HM1	0.873	< 0.001	0.804	0.884	0.878
	HM2	0.901	< 0.001			
	HM3	0.915	< 0.001			
Perceived Ease of Use	PEOU1	0.934	< 0.001	0.804	0.836	0.778
	PEOU2	0.870	< 0.001			
Perceived Risk	PR1	0.785	< 0.001	0.581	0.811	0.768
	PR2	0.683	0.003			
	PR3	0.783	< 0.001			
	PR4	0.792	< 0.001			
Perceived usefulness	PU1	0.778	< 0.001	0.593	0.846	0.846
	PU2	0.673	< 0.001			
	PU3	0.777	< 0.001			
	PU4	0.857	< 0.001			
	PU5	0.756	< 0.001			
Social Influence	SI1	0.623	< 0.001	0.596	0.701	0.701
	SI2	0.850	< 0.001			
	SI3	0.824	< 0.001			

**Table 3:** Fornell–Larcker criterion

	At	BI	HM	PEOU	PR	PU	SI
At	0.862						
BI	0.804	0.933					
HM	0.79	0.702	0.897				
PEOU	0.345	0.32	0.295	0.903			
PR	0.171	0.114	0.149	0.152	0.762		
PU	0.644	0.603	0.556	0.237	0.014	0.77	
SI	0.487	0.517	0.586	0.273	0.311	0.455	0.772

**Table 4:** HTMT and Confidence Interval

	At	BI	HM	PEOU	PR	PU	SI
At							
BI	0.926 (0.880, 0.974)						
HM	0.889 (0.833, 0.939)	0.805 (0.725, 0.881)					
PEOU	0.408 (0.245, 0.575)	0.384 (0.205, 0.570)	0.337 (0.198, 0.492)				
PR	0.198 (0.120, 0.397)	0.131 (0.070, 0.309)	0.173 (0.095, 0.351)	0.185 (0.095, 0.373)			
PU	0.732 (0.641, 0.813)	0.694 (0.599, 0.786)	0.628 (0.471, 0.767)	0.307 (0.189, 0.458)	0.161 (0.159, 0.335)		
SI	0.632 (0.470, 0.766)	0.669 (0.573, 0.798)	0.757 (0.653, 0.877)	0.383 (0.208, 0.567)	0.466 (0.232, 0.684)	0.581 (0.418, 0.749)	

the bias-corrected, bootstrapped confidence intervals are listed in parentheses

**Fig. 1:**
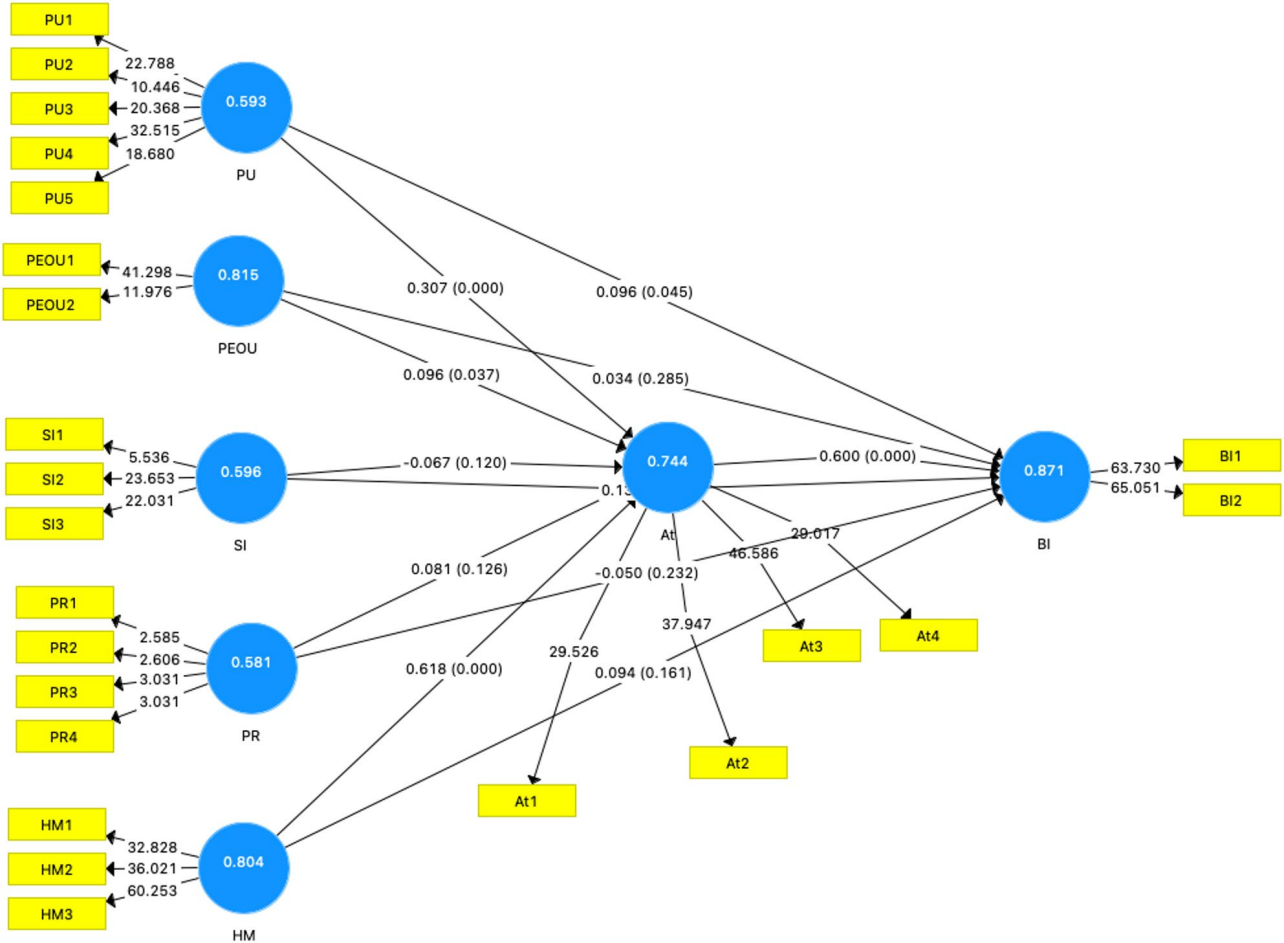
Path coefficient analysis. The numbers in both the inner and outer models are the path coefficient values (p-values) and the numbers inside the variables are the AVE

**Fig. 2:**
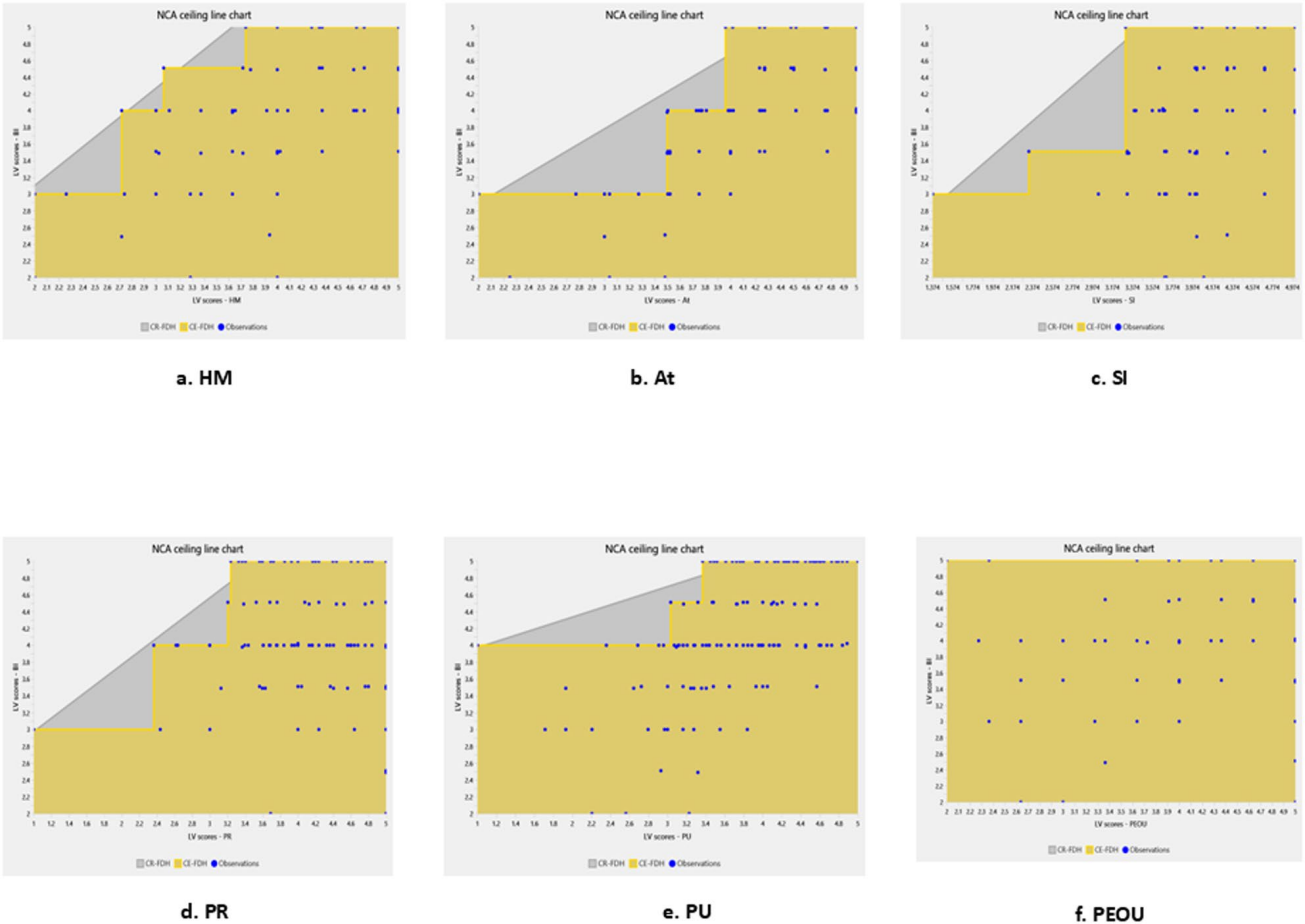
Scatter plots for the ceiling zones exerted by the 6 independent variables (HM = Hedonic Motivation; At = Attitude; SI = Social Influence; PR = Perceived Risk; PU = Perceived Usefulness and PEOU = Perceived Ease of Use) on Behavioral Intention

Subsequently, the structural model showed an overall substantial model fit with R^2^ = 0.68. All variance inflation factor (VIF) values were below the critical threshold of 5 [20], indicating no multicollinearity concerns in the structural model and that common method variance was unlikely to be a major threat (see Table 5). With Q^2^_predict_ values > 0 and lower RMSE values for all variables compared to the naïve linear regression (LM) benchmark, the model demonstrated high predictive power [24]. Among the 6 independent variables, only 3 variables had significant influence on behavioral intention (BI) (Fig. 1). Attitude (At) exerted the strongest influence on behavioral intention (BI) (path coefficient = 0.600) followed by social influence (SI) (path coefficient = 0.133) and perceived usefulness (PU) (path coefficient = 0.096) (see Table 5 for detailed results and the scatter plots in Fig. 2 on showing the ceiling zones of each of the variables exerted on BI). Correspondingly, attitude showed a medium to substantial effect size (f^2^) of 0.337; whereas social influence and perceived usefulness had small to moderate effect sizes with f^2^ values of 0.031 and 0.015 respectively [20]. These three significant variables (i.e., attitude, social influence, and perceived usefulness), were also found to be significant necessary conditions as well with d values of 0.384, 0.310 and 0.183 respectively.

**Table 5:** Combined structural model from SEM and NCA results

	Structural Model from SEM						NCA		Interpretation
	Inner model VIF	Path coefficients	Standard deviation	T statistics	Effect size (f^2^)	*p*-values	Effect size (d)	Permutation *p*-value	
At ◊ BI	3.340	0.600	0.089	6.767	0.337	< 0.001	0.384	0	Hypothesis HI was supported. Attitude is both a necessary and sufficient condition for behavioral intention
HM ◊ BI	3.122	0.094	0.099	0.954	0.009	0.170	0.235	0	Hypothesis H2 was not supported. Hedonic motivation is a necessary but not sufficient condition for behavioral intention
PEOU ◊ BI	1.160	0.034	0.062	0.545	0.003	0.293	0	-	Hypothesis H3 was not supported. Perceived ease of using ChatGPT is neither necessary nor sufficient condition for behavioral intention
PR ◊ BI	1.165	-0.050	0.07	0.725	0.007	0.234	0.299	0.029	Hypothesis H4 was not supported. Perceived risk is a necessary but not a sufficient condition for behavioral intention
PU ◊ BI	1.861	0.096	0.056	1.708	0.015	0.044	0.183	0.025	Hypothesis H5 was supported Perceived usefulness is both a necessary and sufficient condition for behavioral intention
SI ◊ BI	1.767	0.133	0.068	1.945	0.031	0.026	0.310	0.031	Hypothesis H6 was supported. Social influence is both a necessary and sufficient condition for behavioral intention
		Other path coefficient analysis							
HM ◊ At	1.846	0.618	0.064	9.628	0.691	< 0.001			Hypothesis H7 was supported. Hedonic motivation had a significant influence on attitude
PEOU ◊ At	1.129	0.096	0.053	1.802	0.027	0.036			Hypothesis H8 was supported. Perceived ease of use had a significant influence on attitude
PR ◊ At	1.143	0.081	0.067	1.207	0.019	0.114			Hypothesis H9 was not supported. Perceived risk did not have a significant influence on attitude
PU ◊ At	1.545	0.307	0.064	4.767	0.204	< 0.001			Hypothesis H10 was supported. Perceived usefulness had a significant influence on attitude
SI ◊ At	1.752	-0.067	0.060	1.113	0.008	0.133			Hypothesis H11 was not supported. Social influence did not have a significant influence on attitude

Although hedonic motivation and perceived risk did not show significant effects on behavioral intention in the SEM analysis, the complementary NCA results showed that these two variables were meaningful (d ≥ 0.1) and significant (*p* < 0.05) necessary conditions for behavioral intention. In contrast, perceived ease of use did not meet the threshold as a significant necessary condition. Furthermore, hedonic motivation (HM), perceived ease of use (PEOU) and perceived usefulness (PU) were shown to have significant influence on attitude with path coefficients of 0.618, 0.096, 0.307 respectively.

Conceptually, this is further supported by the bottleneck analysis which reveals the threshold levels of the necessary conditions that must be met to achieve a given level of behavioral intention (Table 6). To achieve a full (100%) level of behavioral intention, all these 5 necessary conditions (attitude, hedonic motivation, perceived risk, perceived usefulness and social influence) must be present. At the 50% level of behavioral intention, 4 of these necessary conditions must be present, except perceived usefulness, implying that perceived usefulness has the smallest effect size, with d = 0.183, among these 5 necessary conditions.

**Table 6:** Bottleneck analysis of constraints imposed on behavioral intention (NN = not necessary)

	BI	At	HM	PEOU	PR	PU	SI
0%	2.000	NN	NN	NN	NN	NN	NN
10.000%	2.300	NN	NN	NN	NN	NN	NN
20.000%	2.600	NN	NN	NN	NN	NN	NN
30.000%	2.900	NN	NN	NN	NN	NN	NN
40.000%	3.200	3.498	2.717	NN	2.363	NN	2.338
50.000%	3.500	3.498	2.717	NN	2.363	NN	2.338
60.000%	3.800	3.498	2.717	NN	2.363	NN	3.304
70.000%	4.100	3.960	3.063	NN	3.204	3.033	3.304
80.000%	4.400	3.960	3.063	NN	3.204	3.033	3.304
90.000%	4.700	3.960	3.740	NN	3.237	3.368	3.304
100.000%	5.000	3.960	3.740	NN	3.237	3.368	3.304

## Discussion

From the PLS-SEM analysis, attitude emerged as the strongest predictor of behavioral intention to adopt ChatGPT in education. This aligns with Ajzen and Fishbein’s principle of compatibility [25], which states that the extent to which an attitude predicts behavioral intention depends on the degree of alignment between the behavior’s target, action, context, time or cost associated with performing this action. In this study, intention was specifically tied to using ChatGPT (“target”) by engaging with prompts (“action”) for learning purposes (“context”) during study moments such as revision (“time”), which likely strengthened the attitude–intention relationship.

Similarly, perceived usefulness also showed a significant positive influence on attitude and behavioral intention. This relationship may be explained through the Expectancy-Value Theory [26]. This theory posits that motivation to perform a task is dependent on one’s expectation of success as well as the subjective value he or she assigns to the task. Four types of subjective value have been described, i.e., (1) attainment value, (2) intrinsic value; (3) utility value and the (4) cost incurred. In this regard, medical students are more inclined to use ChatGPT when they perceive that it is useful to support their academic achievement (utility value), useful in helping them to achieve their professional identity as future doctors (attainment value), and useful in making learning engaging (intrinsic value), even when considering the time and effort involved (cost value).

Social influence also demonstrated a significant positive influence on behavioral intention. This may reflect compliance-based behavior [27] or instrumental conformity [28, 29]. Compliance-based behavior occurs when a student decides to use ChatGPT, not out of personal conviction but in response to the perceived expectations from peers [30]. Such influence of “fear of missing out” or FOMO can be particularly strong in today’s digital academic environments. On the other hand, the significance of social influence in using ChatGPT may also be interpreted through Herbert Kelman’s framework of instrumental conformity [28, 29]. In this context, students may adopt a behavior (i.e., using ChatGPT for academic purposes) due to pragmatic reasons such as participating in group tasks, or staying competitive, even if their personal attitudes remain neutral or skeptical.

By contrast, perceived ease of use did not show a significant direct net effect on behavioral intention, although it had a small but significant positive influence on attitude. A plausible explanation is the ceiling effect. ChatGPT’s conversational interface is already intuitive and accessible even for students with limited technical proficiency. Consequently, differences in ease of use may no longer meaningfully distinguish students’ behavioral intentions. Nevertheless, its marginally significant positive relationship with attitude (At) suggests that PEOU may still exert an indirect influence on behavioral intention through attitude formation.

Complementing the SEM analysis, NCA revealed that attitude, perceived usefulness, and social influence were not only sufficient predictors but also necessary conditions for behavioral intention, indicating that high behavioral intention is unlikely to occur unless these factors reach minimum threshold levels. Practically, this suggests that educational strategies should not merely aim to increase intention through these factors, but should ensure that minimum levels of favorable attitudes, perceived value, and supportive social norms as well.

Importantly, NCA also identified hedonic motivation and perceived risk as necessary but not sufficient conditions. Hedonic motivation therefore functions as a threshold requirement: a minimum level of enjoyment is needed for adoption to become viable, but increasing enjoyment beyond that threshold does not necessarily strengthen intention further.

Drawing on Kahneman’s conceptualization of hedonic experience [31], sustained engagement may depend on present enjoyment, remembered satisfaction from prior use, and anticipated future pleasure. This implies that educators should consciously inject a minimum level of fun and enjoyment into AI-based learning at all times to ensure that it remains engaging and intrinsically rewarding to the students.

Similarly, perceived risk (PR) was identified as another necessary but not sufficient condition, suggesting that a minimal level of risk mitigation must be in place for students to engage safely with ChatGPT, although reducing such risks alone may not significantly increase behavioral intention. This could be due to the fact that generative AI like ChatGPT may produce content that appears credible yet factually inaccurate, i.e., a phenomenon known as AI hallucination [32]. Such risks can be particularly pertinent in medical education, where factual accuracy is paramount. Additionally, the “black box” nature of OpenAI’s model raises privacy and transparency concerns, as users cannot ascertain how confidential data are processed or ethically stored in the generative AI platform. Taken together, because both HM and PR are merely necessary but not sufficient conditions, further increases in enjoyment or improvements in risk mitigation beyond the minimum threshold level would not meaningfully enhance students’ behavioral intention to use ChatGPT. Practically, this means AI-based learning should remain at least moderately enjoyable (e.g., with interactive case prompts) and supported by clear risk safeguards (e.g., emphasis on hallucination awareness). But once these baseline conditions are secured, greater emphasis, energy and resources should be directed toward strengthening factors that are both necessary and sufficient (e.g., attitude, perceived usefulness, and social influence), as additional effort spent solely on enjoyment or risk mitigation is unlikely to produce proportional gains in intention.

This study has several pertinent limitations that should be acknowledged. First, as the sample was limited to a single cohort of pre-clinical Year 2 medical students from one institution, this might have limited the generalizability of findings to students in clinical years as well as students from other programs or educational settings. In this regard, a multi-center study across multiple year groups is therefore needed to confirm the generalizability of these results. Second, its cross-sectional design captures the students’ perceptions and behavioral intentions at a single time point only and would have prevented the capturing of causal relationship changes as the students’ experiences with ChatGPT evolves over a longer time period. Third, data were collected through self-administered questionnaires. This would have rendered the responses susceptible to social desirability and Hawthorne effects, where students might have overstated their positive attitudes toward ChatGPT. Fourth, the relatively homogenous Year 2 medical student cohort high baseline digital literacy and familiarity with these AI tools, could have reduced variability in perceived ease of use and contributed to the non-significant role of PEOU in this study. Finally, as the data were collected in December 2024, these findings reflect students’ perceptions of a specific ChatGPT version available at that time. Given the rapid iterations and frequent updates to the GPT model, newer versions may differ substantially in accuracy and functionality, thus potentially shifting perceptions and attitudes in future contexts.

## Conclusion

This study is believed to be among the first in medical education to integrate PLS-SEM and NCA to determine medical students’ intention to adopt ChatGPT for learning. The findings reveal that attitude, perceived usefulness, and social influence are both necessary and sufficient conditions, while hedonic motivation and perceived risk are necessary but not sufficient, conditions. Importantly, if PLS-SEM were used alone, hedonic motivation and perceived risk might be interpreted as unimportant. However, NCA demonstrates that both of these factors remain indispensable prerequisites that must reach a minimum level for high behavioral intention to be feasible. By combining both PLS-SEM and NCA, this approach enhances the richness and granularity of interpretation by offering a more nuanced understanding of the factors shaping AI adoption in medical education.

## Supplementary Information

Supplementary Material 1.

## Data Availability

The datasets generated and/or analyzed during this study are not publicly available in order to protect the privacy of our medical students as participants of the study but are available from the corresponding author on reasonable request.

## Abbreviations

AI: Artificial Intelligence
At: Attitude
AVE: Average Variance Extracted
BI: Behavioral Intention
CE: Ceiling Envelopment
CR: Ceiling Regression
d: Effect Size (NCA)
f²: Effect Size
FDH: Full Disposal Hull
FOMO: Fear of Missing Out
HM: Hedonic Motivation
HREC: Human Research Ethics Committee
LM: Linear Model
NCA: Necessary Condition Analysis
NN: Not necessary
PEOU: Perceived Ease of Use
PLS-SEM: Partial Least Squares–Structural Equation Modeling
PR: Perceived Risk
PU: Perceived Usefulness
Q²: Predictive Relevance (Q-squared)
RMSE: Root Mean Squared Error
SEM: Structural Equation Modeling
SI: Social Influence
TAM: Technology Acceptance Model
UNIMAS: Universiti Malaysia Sarawak
UTAUT: Unified Theory of Acceptance and Use of Technology
UTAUT2: Unified Theory of Acceptance and Use of Technology 2
VIF: Variance Inflation Factor
VRS: Varying Return to Scale

## References

[1] Gupta N, Khatri K, Malik Y, Lakhani A, Kanwal A, Aggarwal S, et al. Exploring prospects, hurdles, and road ahead for generative artificial intelligence in orthopedic education and training. BMC Med Educ. 2024;24(1):1544. 10.1186/s12909-024-06592-8 PMC11681633 39732679

[2] Zhang Q, Huang Z, Huang Y, Wang G, Zhang R, Yang J, et al. Generative AI in medical education: feasibility and educational value of LLM-generated clinical cases with MCQs. BMC Med Educ. 2025;25(1):1502. 10.1186/s12909-025-08085-8 PMC12560302 41146115

[3] Lee QY, Chen M, Ong CW, Ho CSH. The role of generative artificial intelligence in psychiatric education– a scoping review. BMC Med Educ. 2025;25(1):438. 10.1186/s12909-025-07026-9 PMC11938615 40133891

[4] Moskovich L, Rozani V. Health profession students’ perceptions of ChatGPT in healthcare and education: insights from a mixed-methods study. BMC Med Educ. 2025;25(1):98. 10.1186/s12909-025-06702-0 PMC11748239 39833868

[5] Davis FD, Perceived, Usefulness. Perceived Ease of Use, and User Acceptance of Information Technology. Manage Inform Syst Q. 1989;13(3):319–40.

[6] Venkatesh V, Morris MG, Davis GB, Davis FD. User Acceptance of Information Technology: Toward A Unified View. Manage Inform Syst Q. 2003;27(3):425–78.

[7] Venkatesh V, Thong JYL, Xu X. Consumer Acceptance and Use of Information Technology: Extending the Unified Theory of Acceptance and Use of Technology1. Manage Inform Syst Q. 2012;36(1):157–78.

[8] Albayati H. Investigating undergraduate students’ perceptions and awareness of using ChatGPT as a regular assistance tool: A user acceptance perspective study. Computers Education: Artif Intell. 2024;6:100203.

[9] Bhat MA, Tiwari CK, Bhaskar P, Khan ST. Examining ChatGPT adoption among educators in higher educational institutions using extended UTAUT model. J Inform Communication Ethics Soc. 2024;22(3):331–53.

[10] Salifu I, Arthur F, Arkorful V, Abam Nortey S, Solomon Osei-Yaw R. Economics students’ behavioural intention and usage of ChatGPT in higher education: a hybrid structural equation modelling-artificial neural network approach. Cogent Social Sci. 2024;10(1):2300177.

[11] Liu F, Chang X, Zhu Q, Huang Y, Li Y, Wang H. Assessing clinical medicine students’ acceptance of large language model: based on technology acceptance model. BMC Med Educ. 2024;24(1):1251. 10.1186/s12909-024-06232-1 PMC11533422 39490999

[12] Sallam M, Salim NA, Barakat M, Al-Mahzoum K, Al-Tammemi AB, Malaeb D, et al. Assessing Health Students’ Attitudes and Usage of ChatGPT in Jordan: Validation Study. JMIR Med Educ. 2023;9:e48254. 10.2196/48254 PMC10509747 37578934

[13] Budhathoki T, Zirar A, Njoya ET, Timsina A. ChatGPT adoption and anxiety: a cross-country analysis utilising the unified theory of acceptance and use of technology (UTAUT). Stud High Educ. 2024;49(5):831–46.

[14] Alqaisi N, Alshwayyat S, Aburumman S, Qassim N, Almasri N, Algroosh F, et al. Assessing ChatGPT adoption in Jordanian medical education: a UTAUT model approach. BMC Med Educ. 2025;25(1):750. 10.1186/s12909-025-07336-y PMC12100967 40405195

[15] Almogren AS, Al-Rahmi WM, Dahri NA. Exploring factors influencing the acceptance of ChatGPT in higher education: A smart education perspective. Heliyon. 2024;10(11):e31887. 10.1016/j.heliyon.2024.e31887 PMC11154614 38845866

[16] Richter NF, Schubring S, Hauff S, Ringle CM, Sarstedt M. When predictors of outcomes are necessary: guidelines for the combined use of PLS-SEM and NCA. Industrial Manage Data Syst. 2020;120(12):2243–67.

[17] Dul J. Necessary Condition Analysis (NCA):Logic and Methodology of Necessary but Not Sufficient Causality. Organizational Res Methods. 2016;19(1):10–52.

[18] Ringle C, Wende S, Becker J-M. SmartPLS 4 Bönningstedt: SmartPLS; 2024 [Available from: https://www.smartpls.com/]

[19] Anderson JC, Gerbing DW. Structural Equation Modeling in Practice: A Review And Recommended Two-Step Approach. Psychol Bull. 1988;103:411–23.

[20] Hair JF, Risher JJ, Sarstedt M, Ringle CM. When to use and how to report the results of PLS-SEM. Eur Bus Rev. 2019;31(1):2–24.

[21] Fornell C, Larcker DF. Evaluating Structural Equation Models with Unobservable Variables and Measurement Error. J Mark Res. 1981;18(1):39–50.

[22] Henseler J, Ringle CM, Sarstedt M. A new criterion for assessing discriminant validity in variance-based structural equation modeling. J Acad Mark Sci. 2015;43(1):115–35.

[23] Nunnally JC, Bernstein IH. Psychometric theory. 3rd ed. New York: McGraw-Hill.; 1994.

[24] Shmueli G, Sarstedt M, Hair JF, Cheah J-H, Ting H, Vaithilingam S, et al. Predictive model assessment in PLS-SEM: guidelines for using PLSpredict. Eur J Mark. 2019;53(11):2322–47.

[25] Ajzen I, Fishbein M. Attitude-behavior relations: A theoretical analysis and review of empirical research. Psychol Bull. 1977;84(5):888–918.

[26] Wigfield A, Eccles JS. Expectancy–Value Theory of Achievement Motivation. Contemp Educ Psychol. 2000;25(1):68–81. 10.1006/ceps.1999.1015 10620382

[27] Sowden S, Koletsi S, Lymberopoulos E, Militaru E, Catmur C, Bird G. Quantifying compliance and acceptance through public and private social conformity. Conscious Cogn. 2018;65:359–67. 10.1016/j.concog.2018.08.009 PMC6204883 30219289

[28] Kelman HC. Processes of Opinion Change. Pub Opin Q. 1961;25(1):57–78.

[29] Hopper E, Weyman A. Modes of Conformity and Forms of Instrumental Adjustment to Feelings of Relative Deprivation. Br J Sociol. 1975;26(1):66–77. 1122354

[30] Ajzen I. The theory of planned behavior. Organ Behav Hum Decis Process. 1991;50(2):179–211.

[31] Kahneman D, Diener E, Schwarz N. Well-being: The foundations of hedonic psychology. New York, NY, US: Russell Sage Foundation; 1999.

[32] Wu X, Duan R, Ni J. Unveiling security, privacy, and ethical concerns of ChatGPT. J Inform Intell. 2024;2(2):102–15.

