# Supplementary material for: Sufficient and necessary conditions for ChatGPT adoption in medical education: a combined partial least square-structural equation modelling and necessary condition analysis
Source: BMC Med Educ. 2026 Apr 29;26:977. doi: 10.1186/s12909-026-09244-1 (PMC13274007; doi:10.1186/s12909-026-09244-1)
Supplement: Supplementary file 1 — Supplementary Material 1. [file 12909_2026_9244_MOESM1_ESM.docx]

**Supplementary file: Instrument**

**Sufficient and Necessary Conditions for ChatGPT Adoption in Medical Education: A Combined Partial Least Square-Structural Equation Modelling and Necessary Condition Analysis**

**Part A: Sociodemographic data**

| Name: |  |
| --- | --- |
| Gender: |  |
| Age: |  |

**Part B**

**Evaluation of student’s perception of usefulness on ChatGPT in Learning**

Based on these statements, how do you perceive the usefulness of ChatGPT used for learning?

Please answer using the following scale

1. Strongly disagree
2. Disagree
3. Neutral
4. Agree
5. Strongly agree

|  |  | **Likert scale** | | | | |
| --- | --- | --- | --- | --- | --- | --- |
|  | **Statement** | **1** | **2** | **3** | **4** | **5** |
| PU1 | ChatGPT improve my work efficiency/learning ability/clinical decision-making ability. |  |  |  |  |  |
| PU2 | ChatGPT give me accurate information and do not need to be verified again. |  |  |  |  |  |
| PU3 | ChatGPT should be included in medical students’ learning category, and I would recommend the large language model to my classmates to help them study or work. |  |  |  |  |  |
| PU4 | ChatGPT effectively meet my needs for course learning, scientific research, or clinical learning. |  |  |  |  |  |
| PU5 | I prefer to use ChatGPT rather than Google, or other search engines or medical references to explain medical topics. |  |  |  |  |  |

**Evaluation of student’s perception on ease-of-use on ChatGPT**

Based on these statements, how do you perceive the ease-of-use of ChatGPT used for learning?

Please answer using the following scale

1. Strongly disagree
2. Disagree
3. Neutral
4. Agree
5. Strongly agree

|  | **Statement** | **1** | **2** | **3** | **4** | **5** |
| --- | --- | --- | --- | --- | --- | --- |
| PEOU1 | Learning to use ChatGPT does not take a lot of time and effort. |  |  |  |  |  |
| PEOU2 | Learning to use ChatGPT does not require much expertise and skills. |  |  |  |  |  |

**Evaluation of student’s perception on the social influence of ChatGPT**

Based on these statements, how do you perceive the social influence of ChatGPT for learning?

Please answer using the following scale

1. Strongly disagree
2. Disagree
3. Neutral
4. Agree
5. Strongly agree

|  | **Statement** | **1** | **2** | **3** | **4** | **5** |
| --- | --- | --- | --- | --- | --- | --- |
| SI1 | The students around me are using ChatGPT. |  |  |  |  |  |
| SI2 | The students around suggested me to use ChatGPT. |  |  |  |  |  |
| SI3 | Students, schools, and society’s views on LLMs influence my use on ChatGPT. |  |  |  |  |  |

**Evaluation of student’s perception on the risk of ChatGPT**

Based on these statements, how do you perceive the risk of ChatGPT for learning?

Please answer using the following scale:

1. Strongly disagree
2. Disagree
3. Neutral
4. Agree
5. Strongly agree

|  | **Statement** | **1** | **2** | **3** | **4** | **5** |
| --- | --- | --- | --- | --- | --- | --- |
| PR1 | If I write course assignments, clinical reports, and academic papers using ChatGPT, I am concerned about being accused of plagiarism or moral problems. |  |  |  |  |  |
| PR2 | I am concerned about the potential security risks of using ChatGPT (e.g., misleading information, and disclosure of research content). |  |  |  |  |  |
| PR3 | I worry that using ChatGPT reduces my ability to think independently. |  |  |  |  |  |
| PR4 | I worry that using ChatGPT can lead to a lack of originality in my course assignments, clinical reports, and academic papers. |  |  |  |  |  |

**Evaluation of student’s perception on the attitudes toward use of ChatGPT**

Based on these statements, how do you perceive the attitudes toward the use of ChatGPT used for learning?

Please answer using the following scale:

1. Strongly disagree
2. Disagree
3. Neutral
4. Agree
5. Strongly agree

|  | **Statement** | **1** | **2** | **3** | **4** | **5** |
| --- | --- | --- | --- | --- | --- | --- |
| At1 | I think it is helpful to use ChatGPT. |  |  |  |  |  |
| At2 | I like to use ChatGPT. |  |  |  |  |  |
| At3 | I have a positive attitude towards using the ChatGPT. |  |  |  |  |  |
| At4 | I expect to continue to use the latest or updated version of ChaGPT in my later studies. |  |  |  |  |  |

**Evaluation of student’s perception on the hedonic motivation toward use of ChatGPT**

Based on these statements, how do you perceive the hedonic motivation of ChatGPT used for learning?

Please answer using the following scale:

1. Strongly disagree
2. Disagree
3. Neutral
4. Agree
5. Strongly agree

|  | **Statement** | **1** | **2** | **3** | **4** | **5** |
| --- | --- | --- | --- | --- | --- | --- |
| HM1 | Overall, using ChatGPT is satisfactory and enjoyable. |  |  |  |  |  |
| HM2 | The response generated by ChatGPT is entertaining and creative. |  |  |  |  |  |
| HM3 | Using ChatGPT is engaging and fun. |  |  |  |  |  |

**Evaluation of student’s perception on the behavioral intention toward use of ChatGPT**

Based on these statements, how do you perceive the behavioral intention of ChatGPT used for learning?

Please answer using the following scale:

1. Strongly disagree
2. Disagree
3. Neutral
4. Agree
5. Strongly agree

|  | **Statement** | **1** | **2** | **3** | **4** | **5** |
| --- | --- | --- | --- | --- | --- | --- |
| BI1 | I would like to recommend ChatGPT to my classmates. |  |  |  |  |  |
| BI2 | I am willing to continue using ChatGPT frequently. |  |  |  |  |  |
